# T-cell immunity: a barrier to Omicron immune evasion

**DOI:** 10.1038/s41392-022-01142-4

**Published:** 2022-08-28

**Authors:** Fei Yu, Wanbo Tai, Gong Cheng

**Affiliations:** 1 College of Life Sciences, Hebei Agricultural University, 071001 Baoding, China; 2 Tsinghua-Peking Joint Center for Life Sciences, School of Medicine, Tsinghua University, 100084 Beijing, China; 3 Institute of Infectious Diseases, Shenzhen Bay Laboratory, 518132 Shenzhen, China

**Keywords:** Adaptive immunity, Vaccines

A recent study published in *Cell* by Naranbhai et al. disclosed that although the extent may vary, T-cell responses induced by infection by or vaccination against the severe acute respiratory syndrome coronavirus 2 (SARS-CoV-2) are cross-reactive toward the Omicron variant in most individuals, highlighting the role T-cell immunity plays in preventing immune evasion by the Omicron and even future variants.^1^

The Omicron variant (B.1.1.529) was defined by WHO on November 26, 2021. Till now, Omicron and its sub-variants from BA.1 to BA.5 have been confirmed and become the dominant circulating strains around the world. The genome of Omicron integrates 36 amino acid mutations in the viral spike protein and 59 non-synonymous mutations in the whole genome. At least 15 of the mutations are in the receptor-binding domain (RBD), the most critical domain of the SARS-CoV-2 for antibody neutralization. As a consequence, neutralizing sera or therapeutic monoclonal antibodies mostly showed reduced efficacy to Omicron compared to the ancestral virus.^2^ This has raised concerns that Omicron might escape from the immunity derived from vaccination or natural infection, so the authors speculated that Omicron-specific substitutions among its genome may weaken specific T-cell receptor binding and escape downstream immune responses. To address this, the authors assessed the cellular responses of volunteers against wild-type and the Omicron variant using PBMCs. In addition, the Delta variant, the previous dominant circulating variant in many countries, was included for the cross-reactive T-cell response evaluation.^3^ With the long-lasting prevalence of SARS-CoV-2 variants and the steady promotion of the vaccination program, prior immunity toward SARS-CoV-2 is common. The authors categorized the immune responses of individuals based on the infection and vaccination status into prior infection, vaccination, both prior infection and vaccination, or boosted vaccination (Fig. 1a).

**Fig. 1:**
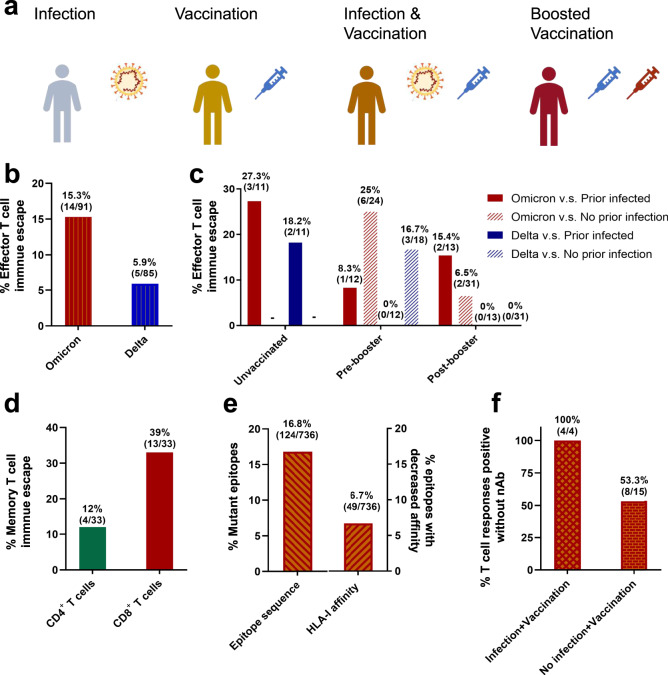
Characteristics of SARS-CoV-2-specific T-cell immune responses towards Omicron variant. **a** Schematic presentation of four main groups of included participants with different SARS-CoV-2 infection or vaccination statuses. Previously infected individuals, solely vaccinated individuals, infected and initially vaccinated individuals, vaccinated and boosted individuals were selected for SARS-CoV-2-specific T-cell immune responses analysis. **b**, **c** Effector T-cell reactivity to the SARS-CoV-2 Omicron and Delta variants. The percentage of individuals whose effector T-cell response was escaped from by the Omicron or Delta variant was analyzed for the general population (**b**) and for the groups with no vaccination, pre-booster, and post-booster vaccination with or without an infection history (**c**). Effector T-cell immune escape was defined as the individual who show a > 50% (0.3 log 10) reduction in effector T-cell response towards antigens from Omicron or Delta. **d**, **e** Memory T-cell immune escape and potential mechanism. CD4^+^ T-cell- and CD8^+^ T-cell-specific carboxyfluorescein succinimidyl ester (CFSE) proliferation assay results showed that CD4^+^ memory T cells had robust proliferative responses against Omicron spike, but escape from CD8^+^ memory T-cell responses were more frequent (**d**). Sequence alignment or binding affinity analysis for HLA class I epitopes predicted by NetMHCpan4.1 confirmed the mutations in epitope sequences and the decrease in HLA class I binding affinity (**e**). **f** Effector T-cell responses to Omicron are preserved even in individuals without detectable neutralizing antibodies of Omicron. A portion of vaccinated individuals with or without prior infection was T-cell response-positive but neutralizing antibody-negative from IFN-γ ELISpot and pseudovirus neutralization assay. The data (**b**–**f**) are reproduced from the original article and supplementary materials. Panels **b**–**f** are adapted from figures and data in the article by Naranbhai et al.^1^

The cross-reactivity of existing anti-SARS-CoV-2 T-cell responses to the Omicron variant was confirmed experimentally by spike-specific IFN-γ ELISpot. However, the T-cell reactivity to Omicron of a unique proportion of infected and/or vaccinated individuals was substantially dampened. Although the number of investigated individuals whose T-cell response was evaded by Omicron was almost three folds compared to that of the Delta variant (Fig. 1b), ELISpot results showed the potential of booster vaccine doses in enhancing T-cell responses. Booster vaccination not only increased the magnitude of circulating effector T-cell responses toward both ancestral SARS-CoV-2 and Omicron by 20-folds but also decreased the immune evasion ratio from 25 to 6.5% in individuals without prior infection (Fig. 1c).

Considering the contribution of CD4^+^ T cells, which correlate to the generation of specific or even neutralizing antibodies, and CD8^+^ cytotoxic T lymphocytes (CTLs), the authors further evaluated the spike-specific CD4^+^ and CD8^+^ memory T-cell responses by revealing the differences in CD4^+^ T cells and CD8^+^ T-cell proliferation assay. The results showed that proliferative CD4^+^, but not CD8^+^, memory T-cell responses were preserved from wild-type to Omicron spike (Fig. 1d). Of note, booster vaccination could enhance the CD8^+^ T-cell cross-reactivity. In order to explore potential mechanisms of the great differences in the specific CD8^+^ T-cell-associated immune responses, the researchers analyzed whether spike protein mutations of the Omicron variant could mediate the escape from CD8^+^ T-cell epitopes recognition by comparing the HLA class I epitopes predicted by NetMHCpan4.1. 736 unique epitopes in the spike proteins of the wild-type and Omicron variant epitopes in total were expected to have substantial binding affinities to HLA-I molecules. Further sequence alignment verified that majority of these epitopes (93.3%, 687/736) were the same or similar between ancestral SARS-CoV-2 and the Omicron variant. For the rest 6.7%, their binding to their associated HLA alleles was susceptible to the mutations in the Omicron variant, and these alleles were in accordance with the HLA haplotypes of individuals with a significant reduction in the effector or memory T-cell responses to the Omicron spike (Fig. 1e). Collectively, the authors obtained critical evidence from both prediction algorithms and experimental data to illustrate that the Omicron spike mutations mediate escape from T-cell responses in an HLA-dependent manner.

Available COVID-19 vaccines, Ad26.COV2.S, mRNA-1273, and BNT162b2 were proved to induce neutralizing antibodies effectively.^4^ However, a portion of individuals, whether infection-naive or prior-infected, could not evoke detectable neutralizing antibodies post vaccination.^5^ In the last part, the authors confirmed that effector T-cell responses to Omicron measured by IFN-γ ELISpot are present in individuals with an undetectable level of neutralization to Omicron (Fig. 1f).

Altogether, the here highlighted study by Naranbhai and colleagues focused on the evaluation of T-cell responses and proposed that the Omicron variant generally did not escape from either effector or memory T-cell responses, except for the CD8^+^ T-cell response in a small portion of individuals. The T-cell responses were preserved because most potential CD8^+^ T-cell epitopes were conserved in the Omicron variant compared to the ancestral wild-type. Booster vaccination can enhance T-cell reactivity to Omicron and further reduce the probability that the Omicron variant escape from T-cell responses. These findings, therefore, support the role of T-cell immune responses in promoting vaccine efficacy and antigen design targeting T-cell immune activation in developing second-generation COVID-19 vaccines.

## References

[1] Naranbhai V (2022). T cell reactivity to the SARS-CoV-2 Omicron variant is preserved in most but not all individuals. Cell.

[2] Cao Y (2022). Omicron escapes the majority of existing SARS-CoV-2 neutralizing antibodies. Nature.

[3] Shi Q, Dong XP (2021). Rapid global spread of the SARS-CoV-2 Delta (B.1.617.2) variant: Spatiotemporal variation and public health impact. Zoonoses.

[4] Naranbhai V (2022). Comparative immunogenicity and effectiveness of mRNA-1273, BNT162b2, and Ad26.COV2.S COVID-19 vaccines. J. Infect. Dis..

[5] Garcia-Beltran WF (2021). COVID-19-neutralizing antibodies predict disease severity and survival. Cell.

